# Laparoscopic Radical Nephrectomy: The New Gold Standard Surgical Treatment for Localized Renal Cell Carcinoma

**DOI:** 10.1100/tsw.2007.153

**Published:** 2007-04-09

**Authors:** Saadettin Yilmaz Eskicorapci, Dogu Teber, Michael Schulze, Mutlu Ates, Christian Stock, Jens J. Rassweiler

**Affiliations:** Department of Urology, SLK-Klinikum Heilbronn, University of Heidelberg, Heidelberg, Germany

**Keywords:** kidney, laparoscopy, nephrectomy, renal cell, carcinoma

## Abstract

We will try to demonstrate that laparoscopic radical nephrectomy could be the new gold standard treatment for renal cell carcinoma with the aid of the current reports exploring the advantages and disadvantages of laparoscopic radical nephrectom overopen surgery.

Reported perioperative outcomes like operating time, blood loss, postoperative analgesia requirement, and length of hospital stay and duration of convalescence had been found to be in favor of laparoscopic radical nephrectomy. Some technical issues like approach of laparoscopic technique (Transperitoneal versus retroperitoneal laparoscopic nephrectomy), removal of dissected specimen and need for lymph node dissection had been also discussed in detail in this review. Besides, oncological safety of laparoscopic radical nephrectomy had been explored. The overall five-year disease free survival rates of laparoscopic radical nephrectomy in recent series were found to be over 90%. All of the series including the present one at least confirmed the oncological efficacy of LRN as compared with open surgical approach.

The contemporary review of the literature documents clearly demonstrated the perioperative benefits of laparoscopy compared to the open approach. Nevertheless, the development, however, more safe and reliable technique in laparoscopy is necessary for tumor extraction. Recent studies confirmed the long-term similar cancer control results of laparoscopic radical nephrectomy with open surgery. Despite some technical modifications by the different groups, it can be stated that laparoscopic radical nephrectomy is the new gold standard treatment modality for patients with localized renal cell carcinoma.

## INTRODUCTION

The profile of patients seeking treatment for renal cell carcinoma has been changed because of the fact, that more incidental renal tumors are diagnosed. Consequently, treatment strategies with more conservative approaches (i.e. high-intensity focus ultrasound, radio-frequency ablation, cryotherapy and laparoscopy) used for the treatment of localized renal cell carcinoma nowadays. Nevertheless, surgical removal is still considered to be the most significant procedure in the management of renal cell carcinoma.

Open radical nephrectomy has been the standard therapeutic modality for localized renal cell carcinoma since Robson first reported it in 1963[1] During the last ten years however open surgery has increasingly been replaced by the laparoscopic approaches. At the beginning of the decade, Clayman et al. pioneered laparoscopic nephrectomy, when they removed a renal oncocytoma in 1990[2]. In the early experiences, the operative time was 5–8 h, and the estimated blood loss was often over 500 ml[3] Nonetheless even in the early series, the postoperative hospital stay and time to full convalescence were significantly shorter than those of the open radical nephrectomy[4,5,6] Currently numerous experiences world wide have demonstrated very good surgical and perioperative results with laparoscopic radical nephrectomy (LRN) and at least comparable or - usually better in many aspects - than the open surgery[3] Besides, recently published series with long term follow up show now a similar oncological results compared to the open counterpart[7,8]. LRN is nowadays regarded as a standard treatment in many clinics worldwide. Besides, nowadays 80% of the urologists offer LRN to their patients with localized renal cell carcinoma[9].

In the present review, we will discuss the important advantages and disadvantages of LRN over open surgery. Moreover, oncological efficacy will be explored wit the aid of the current reports that show the long-term oncological efficacy. Overall, we will try to demonstrate that (LRN) could be the new gold standard treatment for renal cell carcinoma (RCC).

## PATIENTS AND METHODS

Since 1992, we performed 100 laparoscopic radical nephrectomies in 98 patients (58 male, 40 female) with localized renal cell carcinoma in the department of Urology at the SLK–Klinikum in Heilbronn. All relevant perioperative data were recorded, concerning operative time; complications, conversion and reintervention rate as well as hospital stay (Table 1). The majority was pT1 tumors; there had been two bilateral renal cell carcinomas in patients under dialysis (Table 1).

## RESULTS

The operating time averaged 135 (90-410) minutes; there was no difference whether a transperitoneal (n = 20) or retroperitoneal (n= 80) approach was used (Table 1). In 28 cases the specimen was entrapped in an organ bag (LapSac$^{R}$, Cook-Europe) and retrieved after digital morcellation, whereas in 72 instances the intact organ was removed via a 6-8 cm incision in the lower abdomen. In five cases, this incision was also used for manual assistance during the procedure. The mean estimated blood loss was 140 (100 - 700) cc. There was no conversion to open surgery.

We observed one bleeding from the surface of the spleen, which could be managed by laparoscopic tamponating using hemostatic gauze (Tachotamp$^{R}$, Ethicon, Norderstedt). Another patient developed bleeding from one of the trocar site) 6 hours after a right radical nephrectomy which was controlled by a transcutaneous suture. Two months later the same patient suffered from ileus due to a stenosis of the terminal ileum most probably induced by the aforementioned suture. The patient was successfully treated by a segmental ileal resection. One patient had a pulmonary embolism, which could be managed conservatively. The mean postoperative hospital stay was 7 (4-16) days (Table 1).

**Table 1.**
**Heilbronn Experience with Laparoscopic Radical Nephrectomy –**
*Perioperative Data*

| ***Criteria*** | ***Nephrectomy (RCC)*** |
|---|---|
| *Total number* | *100* |
| *Access* | |
| *-transperitoneal* | *20%* |
| *-retroperitoneal* | *80%* |
| *Specimen retrieval* | |
| *- morcellation* | *28%* |
| *- by incision* | *72%* |
| *Mean Operating time* | *135 min .(90-410)* |
| *Mean Blood loss* | *140 cc. (100-700)* |
| *Conversion to open surgery* | *0* |
| *Complications* | *5,0%* |
| *- bleeding* | *2%* |
| *- pulmonary embolism* | *1%* |
| *- ileal stenosis* | *1%* |
| *Reintervention* | *1%* |
| *Hospital Stay(mean)* | *7 d* |
| *Back to normal activity (mean)* | *21 d* |
| *Pathological results* | |
| *T1* | *72%* |
| *T2* | *13%* |
| *T3a* | *9%* |
| *T3b* | *3%* |
| *Oncocytoma* | *3%* |

The tumor was right sided in 40 (40%) patients, left sided in 56 (56%), and bilateral in two (2%) patients. The tumor was located at the upper pole in 34 (34%), at the central area in 43 (43%) and at the lower pole in 23 (23%) of the cases. Mean tumor size was 5,1 cm (range 0,5 to 8). The pathological examination revealed renal cell carcinoma in 97 (97%) and an oncocytoma in three (3%) specimens. In the renal cell carcinoma group, the tumor stage was pT1 in 72 (72%), pT2 in 13 (13%), pT3a in 9 (3 %), and pT3b in 3 (3%) of the specimens. Since we did not use any morcellator (i.e. Cook-morcellator), the pathologist was able to define the exact pathological staging in all cases. The surgical margins were negative in all cases.

The follow-up time averaged 75 (7- 165) months. Outcomes were determined by local recurrence, regional progression, development of metastases and disease specific survival.(Table 2). There was no port-site metastasis. One patient with a pT2G2 tumor developed a local recurrence and bone metastases 4 years after laparoscopic radical nephrectomy. He died 56 months after the procedure. Another five patients with pT1G3 (n=1), pT2G3 (n=1), pT3aG3 (n=2) and pT3b (n=1) tumor developed pulmonary and bony metastases and died 34 months after surgery. The cumulative overall disease-free survival rate after 5 years is 94 %, revealing 96% for pT1/pT2 and 75% for pT3 tumors.

**Table 2.**
**Heilbronn Experience with Laparoscopic Radical Nephrectomy –-**
*follow-up data*

| *Criteria* | *Radical nephrectomy (RCC)* |
|---|---|
| *Total* | *100* |
| *Mean observation time* | *75 mths.* |
| *Dead of disease* | *6%* |
| *Dead of other causes* | *2%* |
| *Overall survival* | *92 %* |
| *Disease-free survival (5 ys.)* | |
| *- overall* | *94%* |
| *- pT1/pT2* | *96 %* |
| *- pT3* | *75 %* |

## DISCUSSION

### Operative Outcomes of Laparoscopic Radical Nephrectomy

Nowadays, most of the patients with RCC are looking for minimal invasive surgical procedures for their treatment. Several studies have shown a clear advantage of LRN in terms of reduced perioperative morbidity, thus minimal invasiveness. Improvements, including reduced operating time, blood loss, postoperative analgesia requirement, length of hospital stay and duration of convalescence, are clearly beneficial to both the patient and the urologist.

The comparison of complication rate, length of hospital stay, blood loss and a decreasing operating time confirms significant lower perioperative morbidity (Table 3)[8,10-18].

The mean operating time initially reported in the range of 240 minutes decreased in recent publications to 150 minute[12,17,19]. Mean operation time of 135 minutes in the present series confirmed the decreasing operation time's wit increasing experience. Dunn et al. reported a decrease of the operating time by nearly half comparing the first 10 and the last 10 patients who underwent a laparoscopic radical nephrectomy in the same institution[12]. Abbou et al., Gill et al., and Janetschek et al. adopted intact removal of the dissected specimen through an additional incision with an operative time of 2.4–3.1 h[11,16,17,19,20]. Barrett et al. reported that average operative time was 2.7 h, although they used morcellation[10].

**Table 3.**
**World-wide experience of laparoscopic radical nephrectomy related to the perioperative morbidity**

| Author | Patients [n] | Tm size (cm) | Approach | Op. time [h] | Blood loss [ml] | Comp rate | Extraction method | Conversion | Convalescence (weeks) | Hospital stay [days] |
|---|---|---|---|---|---|---|---|---|---|---|
| Barrett | 72 | | All T | 2,9 | n.a. | 11% | M | 8% | NA | 4,4 |
| Abbou | 29 | 4,0 | All R | 2,4 | 100 | 8% | I | 3,4% | NA | 4,8 |
| Dunn | 60 | 5,3 | 3 R 58T | 5,5 | 172 | 34% | M and I | 1,6% | 3,2 | 3,4 |
| Cicco | 50 | 3,9 | All R | 2,3 | 150 | 8% | I | 6% | NA | 6 |
| Ono | 103 | 3,1 | 18R 85T | 4,7 | 254 | 30% | M | 3,4% | NA | NA |
| Chan | 67 | 5,1 | n.a. | 4,2 | 289 | 15% | M and I | 1,5% | NA | 3,8 |
| Gill | 100 | 5,1 | 73R 27T | 2,8 | 212 | 14% | M and I | 2% | 4,2 | 1,6 |
| Janetschek | 121 | | | 2,4 | 154 | 9% | I | 0% | | 6,1 |
| Wille | 125 | 5,1 | All T | 3,3 | 210 | 8% | I | 3,2% | NA | 6 |
| Rassweiler* | 100 | 5,1 | 80R 20T | 2,2 | 135 | 5% | M and I | 0% | 3 | 7 |

present series

M:Morcellation I:Intact removal R:Retroperitoneal T:Transperitoneal

In one of the early series, McDougall et al described the advantage of LRN having decreased need for post-operative analgesics[4]. Time to full convalescence was 25 days in the laparoscopy group, which was statistically significantly shorter than 40 days 75 of the open-surgery group in the same series. In 1997, Ono et al reported that a mean dosage of pentazocine was statistically lower in LRN then open-surgery patients (34 mg vs 63 mg; $p < 0.05$)[5] Full convalescence was observed on the 23rd and 64th days, respectively ($p < 0.002$). Both studies clearly pointed out that laparoscopic radical nephrectomy had a minimally invasive nature.

As far as the duration of the hospital stay was concerned, different authors described a significant advantage of laparoscopy: Gill et al. 1,4 vs. 5,8 days, Abbou et al. 4,8 vs. 9,7 days[11,16]. Average estimated blood loss was reported as 100–300 ml in the current literature (Table 3). The rates of conversion to open surgery were 0–8% due to vascular injury or injury to the viscera. (Table 3) The rate of complication was reported to be 5–34% with differences because of some authors including minor complications and others including major ones. (Table 3) In our series of 100 LRN, mean 140 ml blood loss and 0% conversion rate and 5% complication rates were all proved the decreased perioperative morbidity of LRN.

## Technical Issues on Laparoscopic Radical Nephrectomy

Certain technical issues exist in laparoscopic radical nephrectomy: transabdominal versus retroperitoneal approaches, specimen removal intact in fractionation or morcellation, and lymph node dissection.

**Table 4.**
**Laparoscopic versus open radical nephrectomy-** Review of the literature

| ***Criteria*** | ***Abbou*** | | ***Ono*** | | ***Gill*** | | ***Jeschke*** | | ***Dunn*** | |
|---|---|---|---|---|---|---|---|---|---|---|
| | ***Lap*** | ***Op*** | ***Lap*** | ***Op*** | ***Lap*** | ***Op*** | ***Lap*** | ***Op*** | ***Lap*** | ***Op*** |
| *Patients (n)* | *29* | *29* | *103* | *46* | *34* | *34* | *31* | *34* | *61* | *33* |
| *Tumor size (cm)* | *4.1* | *5.7* | *3.1* | *3.3* | *5.0* | *6.1* | *3.8* | *5.7* | *5.3* | *7.4* |
| *OR-time (min.)* | *145* | *121* | *282* | *198* | *186* | *174* | *125* | *145* | *330* | *144* |
| *Blood loss (ml)* | *100* | *285* | *254* | *465* | *98* | *370* | *n.a.* | *n.a.* | *172* | *451* |
| *Complication (%)* | *7* | *27* | *n.a.* | *n.a.* | *13* | *24* | *n.a.* | *n.a.* | *34* | *45* |
| *Hospital stay (d)* | *4.8* | *9.7* | *n.a.* | *n.a.* | *1.4* | *5.8* | *6.8* | *11.5* | *3.4* | *5.2* |
| *Follow-up (mths.)* | *15* | *13* | *29* | *39* | *10* | *29* | *n.a.* | *n.a.* | *25* | *27.5* |

## *Open vs Laparoscopic Nephrectomy*

Several studies had been shown the clear advantages of LRN on open radical nephrectomy especially in terms of perioperative morbidity (Table 4)[11,12,14,19,20]. In a multicenter study, Ono et al compared 103 patients operated by laparoscopy (85 transperitoneal and 18 retroperitoneal) with 46 operated by the classic open procedure[14]. The mean blood loss was documented with 254 ml vs. 465 ml, the mean of the patients requiring transfusion were 5% vs. 9% respectively for the two groups. Gill et al compared retrospectively 34 patients operated laparoscopically using a retroperitoneal approach with 34 patients who underwent traditional open methods[19]. They found a mean blood loss of 97,4 ml versus 295,1 ml and a complication rate of 13% vs 24% in his institution for comparable cases. Dunn et al found similar decreases perioperative morbidity results in addition to the similar recurrence results during the followup of the LRN and open surgery groups[12].

In recent studies early immune responses to open and laparoscopic radical nephrectomy operations were compared. Landman et al recently studied various parameters like adrenalin, noradrenalin and cortisol, inflammatory response markers (C-reactive, protein, white blood count and leukocyte count), lymphocytic response markers (CD3, CD4 and CD8), cytokines interleukin-2 and 4, interferon and tumor necrosis factor-), HLA-DR expression and the proliferative response to mitogen stimulation[21]. Authors found no difference in stress response for open and laparoscopic nephrectomy groups.

## Transperitoneal vs Retroperitoneal Laparoscopic Nephrectomy

Recently published articles with prospective randomized series demonstrated no statistically difference in the overall operative morbidity in transperitoneal versus retroperitoneal radical nephrectomies[22-24].

Desai et al reported that both approaches were similar in terms of blood loss, intraoperative and postoperative complications, length of hospital stay, analgesia requirements[22]. Nevertheless it is remarkable that the retroperitoneal group, compared to the transperitoneal approach, was associated with a shorter total or-time (150 versus 207 minutes, p= 0,001), quicker time to control the renal artery (34 versus 91 minutes, p= < 0,0001) and quicker control of the renal vein (45 versus 98 minutes, p= <0,0001).

Nambirajan et al reported that there were no differences in patients' morbidity and technical difficulty for the surgeon between the transperitoneal and retroperitoneal approaches for laparoscopic radical nephrectomy in a prospective, randomized study including 40 patients with stage cT1 and T2 diseases[23]. Gill et al. and Abbou et al. used the retroperitoneal approach and intact removal through an additional incision[11,16]. Ono et al preferred transperitoneal approach since it provides larger working space and preferred retroperitoneal approach in patients with previous severe abdominal surgery[3].

In the meantime, more than ten years after its first description, the technique of retroperitoneal and transperitoneal laparoscopic radical nephrectomy has been standardized fulfilling the principles of a non-touch minimal invasive uro-oncological surgery. Whereas, some authors proposed the retroperitoneal approach, advocating the advantage of earlier control of the renal artery and the reduced need of dissection (i.e. deflection of the colon), some others mentioned the advantage of larger working space in transperitoneal nephrectomy[3,11,16,25,26]. Despite all abovementioned discussions most of the series demonstrated equal efficacy for both techniques and the choice of the retroperitoneal or transperitoneal approach for LRN seemed to depend mostly on the surgical experience of the surgeon.

## Removal of Dissected Specimen

There has been substantial debate on the mode of extraction of renal tumors. Some centers advocate specimen morcellation to avoid an additional incision and to minimize postoperative morbidity, either by using an electric morcellator or by manually fractionating the specimen to maintain better pathological architecture, while some others use intact removal with additional incision. We used both intact removal and adopted fractionation of the specimens which was previously described[27].
Barrett et al. and Clayman et al. adopted a tissue morcellator for removal of the specimen without any incision, which provided the histology of the tumor, but not the exact pathological stage[8,10]. The major concern is pathological interference with morcellation. Some authors suggested that pathological staging could be hampered with morcellation[28] Another risk is tumor spillage from morcellation.

On the contrary to the concerns, Ono et al reported that proper morcellation did not hamper the pathological stage[14]. They have used fractionation of the specimens for patients with less than a 5-cm tumor diameter in whom the tumor mass can be obtained intact. Histopathologic examination was possible of all 93 specimens and indicated six patients having pathological 3a disease. In addition, no damage to the sacks was caused by scissors or the Kelly clamp. Neither seeding of the tumor cells at the port sites nor dissemination in the working space was found in the cases of fractionated specimen removal. On the other hand, some clinics including our clinic still prefer removal of the dissected specimens intact through an additional incision for a complete pathological examination in especially larger tumors[11,15,16,17].

In a recent study, Varkarakis et al stated that, intact extraction and morcellation are both acceptable options for specimen removal, and the choice should depend on surgeon and patient preferences[29]. They also had found no significant difference in operative time, pain or duration of hospital stay, and the quality of life and recovery appear to be the same in these two groups. On the other hand, the fear of portsite seeding and local recurrence after morcellation is not justified by current data and, with proper technique, morcellation is safe[29]. In studies so far, no benefit other than cosmetics has been found for morcellation compared to intact specimen removal.

In a recent review of port-site metastases in urological laparoscopic surgery only four cases were described in conjunction with specimen morcellation for RCC[30]. Although there was no obvious perforation, in two cases unapproved bags were used for morcellation. Three procedures were for stage T1N0 grade 2 tumors[31,32,33] and one for stage T3N0 grade 4 tumor[34] with sarcomatoid elements. In summary, of the four reported cases, seeding appeared to be a result of poor technique in two cases (use of a ‘plastic’ entrapment bag), and in one was attributed to the aggressive nature of the tumor. Double-layered laparoscopy sacks must be used for any damage by the morcellator to prevent port-site metastases.

### *Lymph Node Dissection*

Lymphadenectomy has been reported to have no beneficial therapeutic effects in the treatment of renal cell carcinoma[3]. With the growing experience and standardized technique some clinics are searching the place of laparoscopic lymphadenectomy in high-risk patients.

Ono et al reported the results of ipsilateral para-aortic lymph nodes in 25 patients with renal cell carcinomas of 5 cm or more in diameter[35]. One of the 24 patients had micro-metastatic lymph node disease and they concluded that extended lymph node dissection could be conducted safely by the laparoscopic procedure[35].

## Oncological Results of Laparoscopic Radical Nephrectomy: Long-term Cancer Control

Despite those abovementioned advantages of LRN, concerns about oncological safety were expressed, as the initial series had a limited mean follow-up of only 2 years. Nevertheless, several articles had been published recently in means of long-term follow-up (Table 5)[7,8,12,14,15,16,18,36].

In the present series overall disease-free survival rate after 5 years is found to be 94 %, revealing 96% for pT1/pT2 and 75% for pT3 tumors. Multiple studies have reported the 5-year disease-free and actuarial survival rates of stage T1/2 M0N0 ranging from 91% to 96% and 81% to 95%, respectively[8,14,37,38]. Portis, Stifelman and Saika et al reported 5-year cancer specific survival rates of 98%, 93% and 94% respectively[7,36,39].

In 2002, Portis et al. reported the long-term outcome of 64 patients with localized renal cell carcinoma undergoing laparoscopic radical nephrectomy[7]. The control cohort consisted of 69 patients with localized disease undergoing open radical nephrectomy. A median follow up was 54 months for laparoscopy and 69 months for open surgery patients. Five-year disease-free survival was 92% in laparoscopy patients and 91% in open-surgery patients. Five-year overall survival was 81 and 89%, respectively. No significant differences in oncological efficacy were found between the laparoscopy and open-surgery groups. Chan et al reported similar results of 67 patients with 5 year survival rates of 95% and 86% for LRN and open-surgery, respectively[15].

**Table 5.**
**Worldwide experience with laparoscopic radical nephrectomy –** Oncological aspects

| Author | Patient [n] | pT stage | follow-up [months] | recurrence port site/local/ distant (%) | Disease free survival | | Cancer specific [illegible]ival | |
|---|---|---|---|---|---|---|---|---|
| | | | | | *5 year* | *10 year* | *5 year* | *10 year* |
| *Janetschek `00* | *73* | *T1-T3a* | *13,3* | *0 / 0 / 0* | *n.a.* | | | |
| *Abbou `00* | *41* | *T1-T3b* | *24,7* | *0 / 2 / 0* | *n.a.* | | | |
| *Ono `01 )* | *103* | *-* | *29* | *0 / 1 / 3* | *92 %* | | | |
| | | | | | | *NA* | *NA* | *NA* |
| Chan `01 | *67* | *T1-T3b* | *36* | *0 / 0 / 3* | *95%* | | | |
| *Gill `01* | *100* | *T1-T3b* | *16,1* | *0 / 0 / 2* | *n.a.* | | | |
| | | | | | *92%* | *NA* | *98%* | *NA* |
| *Portis `02* | *54* | *T1-T3b* | *54* | *0 / 1 / 2* | | | | |
| *Ono 2003* | *281* | *T1-T4* | *29* | | *95%* | *NA* | *NA* | *NA* |
| *Saika* | *195* | *T1* | *40* | *0/0/* | *91%* | *NA* | *94%* | *NA* |
| *Wille* | *125* | *T1-T3b* | *23,5* | *0/0/3* | *n.a.* | | | |
| *Permpongkosol '05* | *67* | *T1-T3b* | *73* | *0/0/4* | *94%* | *94%* | *97%* | *97%* |
| | | | | | | *NA* | *NA* | *NA* |
| *Rassweiler** | *100* | *T1-T3b* | *75* | *0 / 2 / 4* | *94 %* | | | |

*present series

Saika et al reported the long-term results of LRN patients. (36) Recurrence free survival rates had been found as 97% at 5 years and 94% at 10 years in pT1aN0/NxM0 patients, 92% at 5 years in pT1bN0M0 patients and 68% at 5 years in pT2N0M0 patients. The cancer-specific patient survival rate was 94% at 5 years and 88% at 10 years in pT1aN0/NxM0 patients, 90% at 5 years in pT1bN0M0patients and 100%at 5 years in pT2N0M0 patients. Authors could not found any significant differences between laparoscopy and open-surgery patients. Final conclusion of the authors was accepting laparoscopic radical nephrectomy as standard treatment modality for patients with T1 and T2 renal cell carcinoma.

Permpongkosol et al recently reported the long term results of LRN series[38]. Their cohort consisted of 67 LRN with median 73 months follow-up and 54 open surgical cases with median 80 months follow-up in the same time period. The 5 and 10-year disease-free survival rates were 98% in patients with cT1 disease and 84% in those with cT2 in LRN group. The 5 and 10-year cancer specific survival rates were 98% in those with cT1 disease and 95% in those with cT2 for the LRN group. The 5 and 10-year disease-free survival rates were 91% in patients with cT1 disease and 77% in those with cT2 in LRN group. The 5 and 10-year cancer specific survival rates were 90% in those with cT1 disease and 84% and 76% in those with cT2 for open surgical cases. Authors stated that the disease-free and cancer specific survival rates of the laparoscopic group compared with that of the open surgery group revealed no significant difference in patients with cT1 and cT2.

The overall five-year disease free survival rates of laparoscopic radical nephrectomy in recent series were found to be over 90%. All of the series at least confirmed the oncological efficacy of LRN as compared with open surgical approach. Our own five-year experience confirms these results concerning the oncological safety of the LRN.

## CONCLUSION

The remarkable increase in laparoscopic radical nephrectomy in last decade is mostly because many patients wants to have less pain and earlier recovery to full convalescence and normal activity. Developing technology and surgical experience has changed LRN from a lengthy procedure to one that is comparable to open surgery. The contemporary review of the literature documents clearly demonstrated the perioperative benefits of laparoscopy compared to the open approach. Nevertheless, the development, however, more safe and reliable technique in laparoscopy is necessary for tumor extraction. Recent studies confirmed the long-term similar cancer control results of laparoscopic radical nephrectomy with open surgery. Despite some technical modifications by the different groups, it can be stated that LRN is the new gold standard treatment modality for patients with localized renal cell carcinoma.

## ACKNOWLEDGEMENT

The authors are grateful to Dr. Z. Kirkali, Editorial Board Member of *TSW Urology* for valuable advice.
